# Granulocyte colony-stimulating factor (G-CSF) transiently suppresses mitogen-stimulated T-cell proliferative response

**DOI:** 10.1038/sj.bjc.6690344

**Published:** 1999-04

**Authors:** E Reyes, I García-Castro, F Esquivel, J Hornedo, H Cortes-Funes, J Solovera, M Alvarez-Mon

**Affiliations:** Medicine/Immune System Diseases Oncology Service, Department of Medicine ‘Principe de Asturias’, University Hospital, Alcalá University, Carretera Madrid-Barcelona, Km 33.600, 28871 Alcalá de Henares, Madrid, Spain; Medical Oncology Department, ‘12 de Octubre’ University Hospital, Madrid, Spain; Amgen, S.A. Barcelona, Spain

**Keywords:** G-CSF, breast cancer, autologous peripheral blood transplantation, T lymphocytes

## Abstract

Granulocyte colony-stimulation factor (G-CSF) is a cytokine that selectively promotes growth and maturation of neutrophils and may modulate the cytokine response to inflammatory stimuli. The purpose of this study was to examine the effect of G-CSF on ex vivo peripheral blood mononuclear cell (PBMC) functions. Ten patients with breast cancer were included in a clinical trial in which r-metHuG-CSF was administrered daily for 5 days to mobilize peripheral blood stem cells. Ten healthy women were also included as controls. Our data show that G-CSF treatment induces an increase in peripheral blood leucocyte, neutrophil, lymphocyte and monocyte counts. We have found a modulation in the percentages of CD19+, CD45+CD14+, CD4+CD45RA+ and CD4+CD45RO+ cells in PBMC fractions during G-CSF treatment. We have also found a significant reduction in the proliferative response of PBMC to mitogenic stimulation that reverted 14 days after the fifth and the last dose of G-CSF. Furthermore, it was not associated with significant changes in the pattern of cytokine production. The mechanism of this immunoregulatory effect is probably indirect since G-CSF receptor has not been found in T lymphocytes. This mechanism and its potential clinical applications remain to be elucidated. © 1999 Cancer Research Campaign


British Journal of Cancer (1999) 80(1/2), 229–235
© 1999 Cancer Research Campaign
Article no. bjoc.1998.0344

### Granulocyte colony-stimulating factor (G-CSF) transiently suppresses mitogen-stimulated T-cell proliferative response

**E Reyes[1], I García-Castro[2], F Esquivel[1], J Hornedo[2], H Cortes-Funes[2], J Solovera[3] and M Alvarez-Mon[1]**

[1]Medicine/Immune System Diseases Oncology Service, Department of Medicine 'Principe de Asturias' University Hospital, Alcalá University, Carretera Madrid-Barcelona, Km 33.600, 28871 Alcalá de Henares, Madrid, Spain; [2]Medical Oncology Department, '12 de Octubre' University Hospital, Madrid, Spain; [3]Amgen, S.A. Barcelona, Spain

**Summary** Granulocyte colony-stimulation factor (G-CSF) is a cytokine that selectively promotes growth and maturation of neutrophils and may modulate the cytokine response to inflammatory stimuli. The purpose of this study was to examine the effect of G-CSF on ex vivo peripheral blood mononuclear cell (PBMC) functions. Ten patients with breast cancer were included in a clinical trial in which r-metHuG-CSF was administrered daily for 5 days to mobilize peripheral blood stem cells. Ten healthy women were also included as controls. Our data show that G-CSF treatment induces an increase in peripheral blood leucocyte, neutrophil, lymphocyte and monocyte counts. We have found a modulation in the percentages of CD19+, CD45+CD14+, CD4+CD45RA+ and CD4+CD45RO+ cells in PBMC fractions during G-CSF treatment. We have also found a significant reduction in the proliferative response of PBMC to mitogenic stimulation that reverted 14 days after the fifth and the last dose of G-CSF. Furthermore, it was not associated with significant changes in the pattern of cytokine production. The mechanism of this immunoregulatory effect is probably indirect since G-CSF receptor has not been found in T lymphocytes. This mechanism and its potential clinical applications remain to be elucidated.

**Keywords:** G-CSF; breast cancer; autologous peripheral blood transplantation; T lymphocytes

Granulocyte colony-stimulation factor (G-CSF) is a cytokine that selectively promotes growth and maturation of neutrophil progenitor cells by interacting with a specific cell surface receptor (G-CSFR) (Dmetri and Griffin, 1991; Avalos, 1996). Recombinant human G-CSF (r-metHuG-CSF, Filgrastim) has been effective in ameliorating neutropenia and reducing the rate of infection in chemotherapy-treated cancer patients, as well as in patients with severe chronic neutropenia (Lieschke and Burgess, 1992; Naparstek, 1996). It can also mobilize progenitor cells from the bone marrow into peripheral blood that can be used for hematopoietic reconstitution (Molineux et al, 1990; Bensinger et al, 1995; Schmitz et al, 1995). In addition to its role in the regulation of granulopoiesis, G-CSF enhances functions of mature neutrophils such as phagocytosis (Roilides et al, 1991), respiratory burst (Sullivan et al, 1993) and microbicidal activity (Vechiarelli et al, 1995). G-CSF has been shown to facilitate the clinical recovery of the nonneutropenic host in several animal models of infection (Dale et al, 1995) and in clinical trials of non-neutropenic patients with pneumonia (Welte et al, 1996).

G-CSF may also modulate the cytokine response to inflammatory stimuli. In mice, G-CSF pre-treatment attenuated the release of tumour necrosis factor alpha (TNF-α) and reduced the mortality induced by endotoxin administration (Gorgen et al, 1992). In one study, healthy volunteers were treated with G-CSF and the blood was collected at several time points to measure cytokine release in response to different stimuli such as phorbol esters and phytohaemagglutinin (PHA). G-CSF administration caused a significant reduction in the release of TNF-α by stimulated whole blood (Hartung et al, 1995).

Recent observations suggest that G-CSF may also regulate the function of T lymphocytes. In a murine model of T-cell-mediated lethal shock induced by superantigens, G-CSF pre-treatment led to a reduction in mortality accompanied by diminished interleukin (IL)-2 production in vivo (Aoki et al, 1995). In a murine model of graft-versus-host disease, G-CSF administration induced an improvement in survival that was associated with a significant decrease in the production of IL-2 and interferon gamma (IFN-γ) by T lymphocytes incubated with alloantigens or mitogens (Pan et al, 1995).

To assess whether G-CSF has an effect on the function of T lymphocytes from humans, we have studied ex vivo the proliferative response to polyclonal T lymphocyte mitogens of peripheral blood mononuclear cells (PBMC) from breast cancer patients treated with G-CSF for peripheral blood progenitor cell (PBPC) mobilization. In these PBMC, we have also studied the cytokine production after mitogenic activation and the distribution of T lymphocyte subsets. Our data showed that G-CSF treatment induces an increase in peripheral blood lymphocyte counts and a significant reduction in the proliferative response of PBMC to mitogenic stimulation which reverted 14 days after the end of treatment with G-CSF. These results were not associated with significant changes in the pattern of cytokine production.

*Received 12 February 1998*
*Revised 30 July 1998*
*Accepted 9 September 1998*

*Correspondence to:* M Alvarez-Mon M

### MATERIAL AND METHODS

### Patients features

Ten patients (mean age 42.5 years, range 29–61) were included in this study after giving informed consent, all had breast cancer and were enrolled in a protocol for PBPC transplantation. Patients were either in high-risk stages II and III breast cancer, or stage IV of complete clinical remission. All patients had received, prior to mobilization, six cycles of FEC adjuvant cancer chemotherapy (5-fluorouracil, 600 mg $m^{-2}$, 4-epirubicin, 75 mg $m^{-2}$ and cyclophosphamide 600 mg $m^{-2}$) which was repeated every 21 days. None of the patients had other significant intercurrent disease (AIDS, congenital immunodeficiency, lymphoma, leukaemia or myelodysplastic syndrome, LES, rheumatoid arthirits, fever or psychiatric disease). No previous treatment with G-CSF, GM-CSF, radiotherapy, systemic corticoids, IFN or IL-2 were administered. Ten age-matched healthy women were used as controls.

### Study design

PBPC were mobilized from the bone marrow to the peripheral blood with G-CSF at a dose of 10 μg $kg^{-1}$ $day^{-1}$ (Filgrastim, Neupogen®, AMGEN, Thousand Oaks, CA, USA and Hoffman-LaRoche, Basel, Switzerland) for 5 consecutive days (Figure 1). Mobilization was started 5 or 6 weeks after the administration of the last FEC chemotherapy course (five patients at the fifth week and five patients at the sixth week). The pheresis procedures were started on fifth day of mobilization and were continued until a minimum of 2.5 million of CD34+ cells $kg^{-1}$ were collected.

Blood samples for this study were collected before the first dose of G-CSF, 24 h after the first dose, 24 h after the fourth dose and 14 days after the fifth and the last dose of treatment (Figure 1). For each patients, the same healthy volunteers were used throughout the study. Blood from healthy donors was withdrawn and simultaneously processed with that of the patients. White blood cell (WBC) counts were assessed at each point of blood collection using a Coulter STKS (Coulter, Krefeld, Germany).

### Cell separation

PBMC were obtained by centrifugation in a Ficoll-Hypaque (Lymphoprep Nyegaard and Co., Oslo, Norway) density gradient as previously described (Boyum, 1968). After counting, cells were resuspended in RPMI-1640 (Whitaker Bioproducts, Walkersville, USA) supplemented with 10% heat-inactivated fetal bovine serum (Biochrom KG, Berlin, Germany), L-glutamine (2 mM; Biochrom KG), HEPES (25 mM; Biochrom KG) and 1% penicillin–streptomycin (Difco Lab, Detroit, MI, USA). This will be referred to as a complete medium. Cell viability, as checked by trypan blue exclusion, was always greater than 95%.

### Staining and FACS analysis

For immunofluorescence, PBMC were incubated with combinations of fluorescein (FITC, green), phycoerythrin (PE, orange) and peridinin chlorophyll protein conjugate (PerCP, red)-labelled monoclonal antibodies (mAbs). These were used in three-colour combinations to define the PBMC preparations and activated cells (FITC/PE/PerCP). The mAbs used in this study were anti-CD19

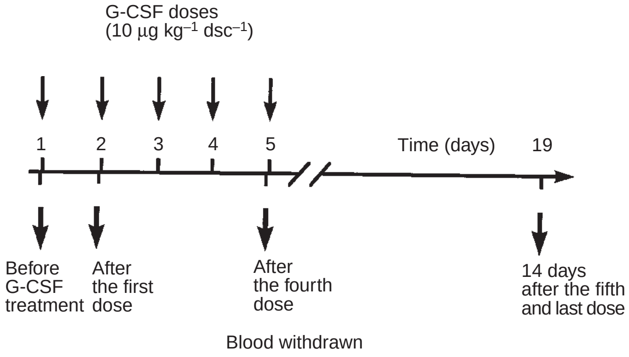

**Figure 1** Summary of the mobilization treatment scheme

(B-cells), anti-CD56 (N-CAM, defines NK cells as CD3–CD56+ and also non-MHC restricted T lymphocytes CD3+CD56+), anti-CD3 (T lymphocytes), anti-CD4 (MHC class II restricted T lymphocytes), anti-CD8 (MHC class I restricted T lymphocytes), anti-HLA-DR (MHC class II molecules), anti-CD25 (p55, IL-2Rα), anti-CD57 (HNK-1, MHC non-restricted cytotoxicity after activation), anti-CD45RO (primed T-cells), anti-CD45RA (unprimed T-cells), anti-IgG, anti-IgM, anti-IgD and anti-CD14 (monocytes). Control studies with unstained cells and cells incubated with isotype-matched irrelevant FITC-, PE- and PerCP-labelled mAbs were performed for each experiment. All mAbs were obtained from Becton Dickinson (Mountain View, CA, USA). Tri-colour immunofluorescent analysis was performed with a FACScan flow cytometer and Lysis II software (Becton Dickinson). For lymphocyte and monocyte population study, a biparametric gate in the FSC-SSC dot plot was drawn around the population as defined by an antigen expression of CD45+CD14– or CD45+CD14+ respectively.

### Proliferation studies

PBMCs (50 000 cells $well^{-1}$) were cultured in complete medium on 96-well, flat-bottomed microtitre plates (Nunc Corporation, Roskilde, Denmark) in the presence or absence of phytohaemagglutinin (PHA, 10 μg $ml^{-1}$; Difco Lab, MI, USA), concanavalin A (Con A, 2 μg $ml^{-1}$; Sigma Chemical Co., MI, USA) and immobilized anti-CD3 (5 μg $ml^{-1}$, Ortho-mune; Orthodiagnostic System). This reagent was tested in dose–response titrations before use. Cultures were incubated at 37°C in a 95% humid atmosphere containing 5% carbon dioxide for 3 and 5 days. DNA synthesis was measured during the last 18 h of the culture period by incorporation of [$^3$H]thymidine (1 μCi; Radiochemical Centre, Amersham, UK) and was expressed in count per minute (cpm). Cultures were performed in triplicate. The standard deviation between replicates was < 10%.

### Measurement of cytokine productions

To measure production of cytokine, $2.5 \times 10^6$ PBMCs from patients and healthy controls were cultured in 1 ml of complete medium in the presence or absence of PHA (10 μg $ml^{-1}$; Difco Lab, MI, USA). Cell cultures were maintained for 72 h in an

 © Cancer Research Campaign 1999

incubator at 37°C in a 95% humid atmosphere containing 5% carbon dioxide. The culture supernatants were then harvested, sterilized by filtration through an 0.22 µm filter (Millipore Company, Bedford, CA, USA) separated into aliquots and immediately stored at –70°C until further use. Cytokine levels in cell culture supernatants were determined in duplicate using the commercially available specific IL-1, IL-6, TNF-α and IFN-γ, enzyme-linked immunosorbent assay (ELISA) kits (Quantikine, RandD Systems, MN, USA), IL-2 ELISA kit (T Cell Diagnostics; Cambridge, MA, USA) and the IL-10 ELISA kit (Bender MedSystems, Vienna, Austria). Each data point is the average of two determinations, neither of which varied within 10% of the final average. Results are expressed in pg $ml^{-1}$. The lower detection limit of the IL-1, IL-2, IL-6, IL-10, TNF-α and IFN-γ test kits was 0.3, 15, 0.7, 3.75, 4.4 and 5 pg $ml^{-1}$ respectively.

### Statistical analysis

Differences in the haematological WBC determination, immune phenotype, proliferation and cytokine release during G-CSF treatment were assessed with analysis of variance (ANOVA) for repeated measurements. Comparison of the measures for the same parameters between patients and healthy controls were tested with Mann–Whitney *U*-test. Correlation between parameters were analysed with Pearson's test. *P*-values were considered significant when less than 0.05. Values reported in the text, tables and figure are means ± standard deviation (s.d.).

### RESULTS

### G-CSF treatment induces an increase in PBMC cells and a transitory switch in certain immune cell subsets

Blood was collected before the first dose of G-CSF (10 µg $kg^{-1}$ $dsc^{-1}$), 24 h after the first dose, 24 h after the fourth dose and 14 days after the fifth and the last dose of G-CSF treatment (Figure 1).

First, the effects of G-CSF treatment in the absolute count of leucocytes in the peripheral blood of patients was studied. No significant differences were found in haematological WBC determinations between healthy controls and patients before G-CSF treatment. However, as can be seen in Table 1, a significant increase in the absolute count of total leucocytes, neutrophils, lymphocytes and monocytes was observed in peripheral blood from patients at 24 h after the first dose of G-CSF. This increase was maintained 24 h after the fourth dose of G-CSF treatment with respect to pre-treatment conditions ($P < 0.05$). The monocyte count increase, 24 h after the fourth dose of G-CSF, was significantly higher than that found after the first dose ($P < 0.05$). However, no significant differences were found in basophile and eosinophile absolute counts at the different time points of the study. Fourteen days after the fifth and the last dose of G-CSF, these changes in cellular counts were no longer significantly different from the values before G-CSF treatment.

Since an increase in lymphocyte counts during G-CSF administration was found, we proceeded to study the distribution of the T lymphocyte subsets in PBMC from these patients (Table 2). G-CSF treatment did not induce significant variations in the percentage of CD3+, CD4+ and CD8+ cells in PBMC from patients. The expression of activation antigen markers HLADR and CD25 (IL-2Rα) by CD3+ cells were not significantly modified by the G-CSF treatment. Twenty-four hours after the fourth dose of G-CSF there was a significant reduction in the percentage of CD4+CD45RA+ naive T-cells associated with a concomitant increase of the CD4+CD45RO+ memory T-cells compared with pre-treatment values ($P < 0.05$). Fourteen days after the fifth and the last dose of G-CSF treatment, this switch in the expression of the RA/RO CD45 isoforms by CD4+ T-cells was reverted. G-CSF treatment did not induce significant modifications in either the percentages of CD56+, CD56+CD3– (Table 2) or of CD3+CD56+, CD3+CD57+, CD57+CD8+CD8+CD3–, CD8+CD3+ (data not shown). In agreement with haematological WBC determinations, previously described, the percentage of CD45+CD14+ monocytes observed 24 h after the fourth dose of G-CSF treatment was significantly higher than that found before treatment ($P < 0.05$).

We did not find significant differences in the expression of the different lymphocyte and monocyte subset markers between PBMC from patients before G-CSF treatment and healthy volunteers.

### G-CSF treatment is associated with a reduced proliferation of PBMC in response to T-cell activation signals

The effects of G-CSF treatment on the proliferative response of PBMC to different T-cell polyclonal mitogens was analysed at 5 days of culture (Figure 2). Before G-CSF treatment, PBMC from patients and healthy volunteers showed similar proliferative response to PHA (patients: 120 196 ± 24 627 cpm; controls: 160 437 ± 32 805 cpm), Con A (patients: 97 986 ± 21 069 cpm; controls: 131 106 ± 27 002 cpm) and anti-CD3 (patients:

**Table 1** Cell recovery ($10^3$ $mm^{-3}$) before, throughout and after G-CSF treatment

| | Pre-treatment | After the first dose | After the fourth dose | Fourteen days after the last dose |
|---|---|---|---|---|
| Leucocytes | 4747 ± 1949 | 30327 ± 10499[a] | 35298 ± 12099[a] | 7080 ± 3318 |
| Neutrophils | 3084 ± 1454 | 27706 ± 10183[a] | 29845 ± 11017[a] | 5373 ± 4000 |
| Lymphocytes | 990 ± 538 | 1439 ± 241[a] | 1944 ± 452[a] | 3318 ± 1000 |
| Monocytes | 375 ± 216 | 1023 ± 417[a] | 2068 ± 891[a,b] | 419 ± 348 |
| Basophiles | 26 ± 13 | 97 ± 60 | 223 ± 166 | 43 ± 43 |
| Eosinophiles | 106 ± 99 | 157 ± 131 | 1114 ± 1000 | 175 ± 70 |

WBC counts were assessed at each point of blood collection using a Coulter STKS (Coulter, Krefeld, Germany). Data are expressed in number of cells × $10^3$ $mm^{-3}$ and are represented as mean ± S.D. [a]$P < 0.05$ versus before G-CSF treatment. [b]$P < 0.05$ versus 24 hours after the first dose of G-CSF.

© Cancer Research Campaign 1999

**Table 2** CD45+CD14– and CD45+CD14+ populations in PBMC cell fractions from patients and healthy volunteers

| Antigens | G-CSF-treated patients | | | | Healthy volunteers |
|---|---|---|---|---|---|
| | Pre-treatment | After the first dose | After the fourth dose | Fourteen days after the last dose | |
| Lymphocytes | | | | | |
| CD45+CD14– | | | | | |
| CD3+ | 57.9 ± 10.9 | 55.9 ± 19.0 | 53.9 ± 19.6 | 57.9 ± 10.2 | 69.5 ± 8.0 |
| CD4+ | 35.3 ± 10.5 | 35.6 ± 16.9 | 29.6 ± 13.3 | 33.6 ± 9.7 | 42.6 ± 10.1 |
| CD8+ | 40.3 ± 8.9 | 36.8 ± 9.2 | 36.3 ± 13.0 | 40.5 ± 8.5 | 37.8 ± 8.4 |
| CD3+HLADR+ | 9.1 ± 7.4[c] | 9.3 ± 6.0 | 8.8 ± 6.1 | 8.9 ± 9.9 | 4.2 ± 3.3 |
| CD3+CD25+ (IL-2Rα) | 8.6 ± 7.6 | 13.3 ± 11.3 | 9.2 ± 9.2 | 8.8 ± 4.2 | 8.4 ± 9.2 |
| CD4+CD45RA+ | 21.4 ± 8.7 | 17.2 ± 13.7 | 12.1 ± 5.4[a] | 14.5 ± 7.1 | 25.6 ± 14.0 |
| CD4+CD45RO+ | 14.1 ± 8.7 | 14.2 ± 8.7 | 24.2 ± 11.9[a] | 15.7 ± 5.1 | 15.3 ± 5.1 |
| CD8+CD45RA+ | 28.1 ± 6.2 | 28.2 ± 8.9 | 23.4 ± 5.4 | 29.7 ± 9.6 | 27.4 ± 6.6 |
| CD8+CD45RO+ | 14.3 ± 7.8 | 11.7 ± 4.3 | 13.0 ± 13.5 | 12.5 ± 4.8 | 12.3 ± 5.6 |
| CD19+ | 11.2 ± 2.9 | 10.5 ± 3.7 | 14.4 ± 4.9[b] | 10.4 ± 3.9 | 8.0 ± 1.8 |
| CD56+ | 37.1 ± 12.3 | 38.7 ± 18.2 | 36.2 ± 18.1 | 36.7 ± 11.5 | 29.0 ± 12.3 |
| CD56+CD3– | 29.8 ± 10.3 | 33.6 ± 18.3 | 30.4 ± 18.6 | 31.7 ± 10.3 | 22.4 ± 9.3 |
| Monocytes | | | | | |
| CD45+CD14+ | 29.5 ± 11.5 | 34.6 ± 10.0 | 43.2 ± 13.0[a] | 25.0 ± 7.8 | 21.9 ± 7.5 |

Blood was collected before the first dose of G-CSF, 24 h after the first dose, 24 h after the fourth dose and 14 days after the fifth and the last dose of treatment. For immunofluorescence, PBMC were incubated with combinations of fluorescein (FITC, green), phycoerythrin (PE, orange) and peridinin chlorophyll protein conjugate (PerCP, red)-labelled mAbs. These were used in three-colour combinations to define the PBMC preparations and activated cells in each tube. Results are expressed in percentage of positive cells and are represented as the mean ± s.d. [a]$P < 0.05$ versus before the G-CSF treatment and 14 days after the fifth and the last dose of G-CSF treatment. [b]$P < 0.05$ versus 24 h of G-CSF treatment and 14 days after the fifth and the last dose of G-CSF treatment. [c]$P < 0.05$ versus healthy controls.

**Table 3** Cytokine production before, throughout and after G-CSF subcutaneous injection at 72 h of culture

| Cytokine release | G-CSF-treated patients | | | | Healthy volunteers |
|---|---|---|---|---|---|
| | Pre-treatment | After the first dose | After the fourth dose | Fourteen days after the last dose | |
| IL-1 | 631 ± 306 | 491 ± 406 | 378 ± 325 | 590 ± 599 | 633 ± 16 |
| IL-2 | 29 ± 31 | 20 ± 15 | 41 ± 36 | 20 ± 18 | 441 ± 283 |
| IL-6 | 1775 ± 339 | 1558 ± 116 | 1469 ± 85 | 1822 ± 328 | 1459 ± 11 |
| IL-10 | 176 ± 163 | 476 ± 609 | 65 ± 63 | 363 ± 378 | 335 ± 49 |
| TNF-α | 660 ± 567 | 392 ± 184 | 538 ± 459 | 397 ± 310 | 459 ± 129 |
| IFN-γ | 435 ± 588 | 701 ± 1134 | 1942 ± 3079 | 1120 ± 1390 | 3833 ± 2823 |

To measure production of cytokine, $2.5 \times 10^6$ PBMCs from patients and healthy volunteers were cultured in 1 ml of complete medium in the presence or absence of PHA (10 µg ml$^{-1}$). Cell cultures were maintained for 72 h in an incubator at 37°C in a 95% humid atmosphere containing 5% carbon dioxide. Results are expressed in pg ml$^{-1}$ and represented as the mean ± s.d.

144 070 ± 28 328 cpm; controls: 147 097 ± 34 445 cpm). Twenty-four hours after the first dose of G-CSF, we observed a non-significant reduction in the PBMC proliferation induced by these mitogenic signals. However, 24 h after the fourth dose of G-CSF this proliferation was further reduced and became significantly lower than the values before G-CSF treatment ($P < 0.05$). This proliferation reduction had partially disappeared 14 days after the fifth and the last dose of G-CSF. There were no significant differences between the proliferative response of PBMC to mitogenic stimulation before G-CSF treatment and 14 days after the fifth and the last G-CSF dose. Similar results were observed after 3 days of culture (data not shown). G-CSF treatment is also associated with a reduced proliferation of purified non-adherent cells in response to T-cell activation signals (manuscript in preparation).

PBMC from patients or controls cultured in absence of mitogenic stimulation did not show any significant proliferation at different times of the study (data not shown). There were no significant differences in the percentages of viable cells found in the PBMC cultures from patients and controls at each time point of the study (data not shown).

The question arises whether this observed decrease in the proliferative response of PBMC from G-CSF-treated patients was

 © Cancer Research Campaign 1999

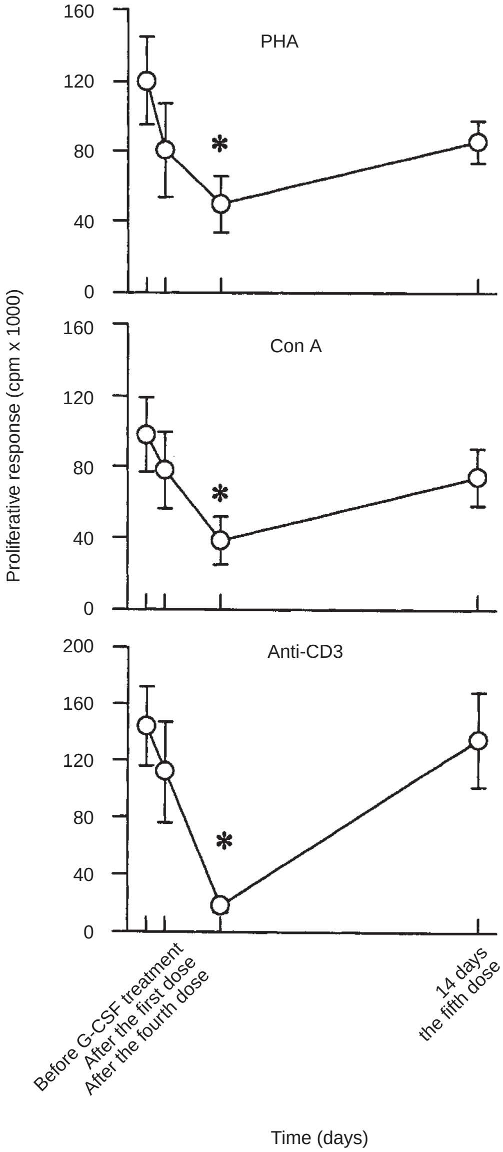

**Figure 2** Proliferative response of PBMC from patients at 5 days of in vitro culture in presence of PHA (10 µg $ml^{-1}$), Con A (2 µg $ml^{-1}$) and anti-CD3 (5 µg $ml^{-1}$. Values are expressed in cpm and represented as the mean ± ES. [a]*P* < 0.05 versus before G-CSF treatment explained by an increase in the number of CD19+ and CD45+CD14+ cells. We analysed the relationship between these phenotypical characteristics and the proliferative response to PHA at 5 days of culture. There were no significant correlations between proliferative response of PBMC to PHA at the different time points of the study and the corresponding percentages of CD3+, CD19+ and CD45+CD14+ cells (data not shown).

### Cytokine production

We have also analysed the cytokine production by PHA-stimulated PBMC (Table 3). No significant differences were observed in the production of IL-1, IL-2, IL-6, IL-10, TNF-α or IFN-γ by PHA-stimulated PBMC from the patients before G-CSF treatment and healthy controls. During G-CSF treatment there were no significant changes in the production of these cytokines by PHA-stimulated PBMC from patients.

We have not found significant differences between the phenotypical and functional parameters analysed in PBMC from patients who received G-CSF treatment 5 weeks or 6 weeks after the administration of the last FEC chemotherapy course (data not shown).

### DISCUSSION

G-CSF stimulates the proliferation and maturation of myeloid progenitor cells to neutrophilic granulocytes (Dmetri and Griffin, 1991; Welte et al, 1996). As expected, G-CSF administered to our patients was associated with a large increase on absolute number of leucocytes and neutrophils from peripheral blood (Sica et al, 1996). As previously described, we observed a rise in monocyte counts induced by G-CSF (Hartung et al, 1995; Pollmacher et al, 1996; Sica et al, 1996). Although it has been reported that lymphocyte production in humans and mice is not affected by G-CSF (Bronchud et al, 1987; Pollmacher et al, 1996), we have observed that G-CSF induces a significant increase in peripheral blood lymphocyte counts. Different G-CSF effects might be involved in the induction of this lymphocytosis. A potential effect of G-CSF in the proliferation and maturation of mononuclear leucocyte precursors may be taken into an account. G-CSF treatment may also modify the tissular distribution of mature lymphocytes as well as their peripheral blood traffic.

As far as we know, this is the first reported observation that G-CSF treatment modulates the proliferative response of PBMC to T-cell mitogens in humans. The proliferation was reduced after the first injection of G-CSF, continued to decline throughout the duration of G-CSF administration and reverted after the end of G-CSF treatment. The mechanism of this immunoregulatory effect is probably indirect since G-CSFR have not been found on T lymphocytes (Shimoda et al, 1992). Proliferation of T-cells in response to antigens and polyclonal mitogens involves the secretion of IL-2 and the binding to its receptor on T lymphocytes (Green and Thompson, 1994; Szamel et al, 1995). It has been observed that G-CSF-treated mice show a reduced production of IL-2 in vivo in response to bacterial superantigens and in mixed lymphocyte reactions in vitro (Aoki et al, 1995). However, in our study, we did not observe either a reduced production of IL-2 or down-regulation in the expression of IL-2Rα (CD25) on PBMC following G-CSF administration. The analysis of our data shows a reduction in the PHA-induced IL-2 production by PBMC from patients before G-CSF treatment, but without statistical significance compared to that of healthy volunteers. Since the proliferative response of patients' PBMC to PHA is normal, the reduced IL-2 production observed in these cellular preparations does not appear to have functional significance. Furthermore, the absence of significant modifications in the IL-2 production by PBMC from patients after G-CSF treatment indicate that the defective proliferative response of these cellular preparations cannot be explained by a impaired production of this cytokine.

Both, in humans and mice, G-CSF administration induces important changes in the release of cytokines from monocytes. In a murine model of endotoxaemia, G-CSF administration was associated with reduced TNF-α serum levels (Gorgen et al, 1992; Kuhns et al, 1995). G-CSF administration to healthy humans caused a reduction in the production of TNF-α and IFN-γ by

© Cancer Research Campaign 1999

lipopolysaccharide-stimulated blood (Hartung et al, 1995). Simultaneously, there was an increase of IL-1 receptor antagonist IL-1ra and soluble TNF receptor (sTNFR) levels induced by lipopolysaccharide 14. Since it is known that several cytokines such as IL-1, IL-6 and IFN-γ play a relevant role in the proliferation of T lymphocytes, we examined whether changes in the production of these mediators could account for the reduced T-cell proliferation. However, we did not observe a decreased production of any of these cytokines by PBMC from G-CSF-treated patients in response to a potent stimulus such as PHA.

Some cytokines, such as IL-10, may attenuate T-cell proliferation by interfering with antigen presentation by macrophages (Nakagomi et al, 1995). We investigated whether up-regulation of IL-10 secretion may explain the observed reduction of T-cell proliferation, but we could not observe any significant production by PBMC from patients during G-CSF treatment.

The activation and growth requirements of CD4+ and CD8+ T lymphocyte subsets, as well as those of their different maturation stages, defined by the expression of RA and RO isoforms of the CD45 antigen, are different (Parnes, 1982; De Jong et al, 1991). Variations in the distributions of these lymphocyte subsets provoke changes in the proliferation of PBMC (Roman et al, 1996). In our study, the percentage of CD4+ and CD8+ T lymphocytes did not change during G-CSF treatment. However, the observed expansion of CD4+CD45RO+ T-cells and concomitant decrease of the naive CD4+CD45RA+ populations may be involved in the functional effects of G-CSF on PBMC.

It remains to be elucidated whether G-CSF may induce lymphocyte anergy–apoptosis. It is known that oxidative stress favours both apoptosis and anergy of T lymphocytes (Buttke and Sandstrom, 1994) and that G-CSF enhances superoxide production by mature neutrophils (Tsuji et al, 1994). The effects of the superoxide production induced by G-CSF treatment on the T lymphocyte functions remain to be defined.

The functional effects of the antigen presentation by accessory cells to T lymphocytes are dependent on different signals (Tsuji et al, 1994; Terashima et al, 1995). These signals regulate the progression of the antigen-primed T lymphocytes to activation–proliferation or anergy – apoptosis pathways. The interaction of different molecules on the surface of accessory cells such as CD80–CD86 with the corresponding counterparts such as CD28 and CTLA-4 on the T lymphocyte surface, regulates the progression of antigen-primed cells to activation or anergy–apoptosis. A potential effect of G-CSF treatment on the expression of these molecules may be to trigger the progression of the T lymphocytes in an anergy–apoptosis pathway.

It may be possible to consider that the immunological modifications observed in G-CSF-treated patients might be part of a recovery phase following chemotherapy instead of due to this cytokine treatment. However, several findings indicate that the immunological variations were due to G-CSF treatment (Sica et al, 1996). Before G-CSF treatment, the phenotypical and functional analysis of PBMC from the patients did not show substantial differences with respect to those found in healthy controls. In all the cases analysed, the modifications were observed after the first, and mainly the fourth, dose of G-CSF. Furthermore, there were no significant differences between the immunological parameters studied on PBMC from the patients analysed 5 or 6 weeks after the last FEC course.

As with the patients in our study, it has become routine clinical practice to administer G-CSF to mobilize from the bone marrow into the PBPC being used for autologous bone marrow transplant. G-CSF has also been used for the mobilization of PBPC in normal volunteers and subsequent transplantation to patients (Welte et al, 1996). It is intriguing that allogeneic PBPC transplantation is not associated with an incidence of graft-versus-host disease higher than in conventional bone marrow transplantation despite the fact that PBPC contains tenfold more T lymphocyte than bone marrow infusions (Pan et al, 1995). Our finding, which indicates a reduction of T-cell proliferation following G-CSF injection, may explain this observation. The reported improvement of inflammatory bowel disease by G-CSF (Roe et al, 1992) may also be related to this effect on T lymphocyte activation.

In conclusion, we have shown that G-CSF causes a reversible decrease in T lymphocyte activation in humans. The mechanism of this effect and its potential clinical applications remains to be elucidated.

### ACKNOWLEDGEMENTS

This work was partially supported by a grant from Comisión Interministerial de Ciencia y Tecnología SAF93-0925-C02-02 and SAF96-0201-C02-01.

### REFERENCES

Aoki Y, Hiromatsu K, Kobayashi N, Hotta T and Saito H (1995) Protective effect of granulocyte colony-stimulating factor against T-cell mediated lethal shock triggered by superantigens. *Blood* **86**: 1420–1427

Avalos B (1996) Molecular analysis of the granulocyte colony-stimulating factor receptor. *Blood* **88**: 761–777

Bensinger WI, Weaver CH, Appelbaum FR, Rowley S, Demirer T, Sanders J, Storb R and Buckner CD (1995) Transplantation of allogeneic peripheral blood stem cells mobilized by recombinant human granulocyte colony-stimulating factor. *Blood* **85**: 1655–1658

Boyum AJ (1968) Isolation of mononuclear cell and granulocytes from human blood. *Scan J Clin Lab Invest* **21**: 77–89

Bronchud MH, Scarffe JH, Thatcher N, Crowther D, Souza LM, Alton NK, Testa NG and Dexter TM (1987) Phase I/II study of recombinant human granulocyte colony-stimulating factor in patients receiving intensive chemotherapy for small cell lung cancer. *Br J Cancer* **56**: 809–813

Buttke TM and Sandstrom PA (1994) Oxidative stress as a mediator of apoptosis. *Immunol Today* **15**: 7–10

Dale DC, Liles C, Summer WR and Nelson S (1995) Review: Granulocyte colony-stimulating factor-role and relationship in infection diseases. *J Infect Dis* **172**: 1061–1075

De Jong R, Brouwer M, Miedema F and Van Lier RAW (1991) Human CD8+ T lymphocytes can be divided into CD45RA+ and CD45RO+ cells with different requirements for activation and differentiation. *J Immunol* **146**: 2088–2093

Dmetri GD and Griffin JD (1991) Granulocyte colony-stimulating factor and its receptor. *Blood* **78**: 2791–2808

Gorgen I, Hartung T, Leist M, Niehorster M and Tiegs G (1992) Granulocyte colony-stimulating factor treatment protects rodents against lipopolysaccharide-induced toxicity via suppression of systemic tumor necrosis factor a. *J Immunol* **149**: 918–924

Green JM and Thompson CB (1994) Modulation of T cell proliferative response by accessory cell interactions. *Immunol Res* **13**: 234–243

Hartung T, Docke WD, Gantner F, Krieger G, Sauer A, Stevens P, Volk HD and Wendel A (1995) Effect of granulocyte colony-stimulating factor treatment on ex vivo blood cytokine response in human volunteers. *Blood* **85**: 2482–2489

Kuhns DB, Alvord WG and Gallin JI (1995) Increased circulating cytokines, cytokine antagonists, and E-selectin after intravenous administration of endotoxin in humans. *J Infect Dis* **171**: 145–152

Lieschke GJ and Burgess AW (1992) Granulocyte colony-stimulating factor and granulocyte-macrophage colony-stimulating factor. *N Engl J Med* **327**: 99–106

Molineux G, Podja Z, Hampson I, Lord B and Dexter T (1990) Transplantation potential of peripheral blood stem cells induced by granulocyte colony-stimulating factor. *Blood* **76**: 2153–2158

Nakagomi H, Pisa P, Pisa EK, Yamamoto Y, Halapi E, Backlin K, Juhlin C and Kiessling R (1995) Lack of interleukin-2 (IL-2) expression and selective

© Cancer Research Campaign 1999

expression of IL-10 mRNA in human renal cell carcinoma. *Int J Cancer* **63**: 366–371

Naparstek E (1996) Granulocyte colony-stimulating factor, congenital neutropenia, and acute myeloid leukemia. *N Engl J Med* **333**: 516–518

Pan L, Delmonte J Jr, Jalonen CK and Ferrara JL (1995) Pretreatment of donor mice with granulocyte colony-stimulating factor polarizes donor T lymphocytes toward type-2 cytokine production and reduces severity of experimental graft-versus-host disease. *Blood* **86**: 4422–4429

Parnes JR (1982) Molecular biology and function of CD4 and CD8. *Adv Immunol* **44**: 265–312

Pollmacher T, Korth C, Mullington J, Schreiber W, Sauer J, Vedder H, Galanos C and Holsboer F (1996) Effects of granulocyte colony-stimulating factor on plasma cytokine and cytokine receptor levels and on the in vivo host response to endotoxin in healthy men. *Blood* **87**: 900–905

Roe TF, Coates TD, Thomas DW, Miller JH and Gilsanz V (1992) Brief report: treatment of chronic inflammatory bowel disease in glycogen storage disease type Ib with colony-stimulating factors. *N Engl J Med* **326**: 1666–1669

Roilides E, Walsh T, Pizzo PA and Rubin M (1991) Granulocyte colony-stimulating factor enhances the phagocytic and bactericidal activity of normal and defective human neutrophils. *J Infect Dis* **163**: 579–583

Roman LI, Manzano L, De la Hera A, Abreu L, Rossi I and Alverez-Mon M (1996) Expanded CD4+CD45RO+ phenotype and defective proliferative response in T lymphocytes from patients with Crohn's disease. *Gastroenterology* **110**: 1008–1019

Schmitz N, Dreger P, Suttorp M, Rohwedder EB, Haferlach T, Loffler H, Hunter A and Russell NH (1995) Primary transplantation of allogeneic peripheral blood progenitor cells mobilized by filgrastim (granulocyte colony-stimulating factor). *Blood* **85**: 1666–1672

Shimoda K, Okamura S, Harada N and Niho Y (1992) Detection of the granulocyte colony-stimulating factor receptor using biotinylated granulocyte colony-stimulating factor: presence of granulocyte colony-stimulating factor receptor on CD34-positive hematopoietic progenitor cells. *Res Exp Med* **192**: 245–255

Sica S, Rutella S, Di Mario A, Salutari P, Rumi C, Ortu la Barbera E, Etuk B, Menichella G, D'Onofrio G and Leone G (1996) rhG-CSF in healthy donors: mobilization of peripheral hemopoietic progenitors and effect on peripheral blood leukocytes. *J Hemother* **5**: 391–397

Sullivan GW, Carper HT and Mandell GL (1993) The effect of three human recombinant hematopoietic growth factors (granulocyte-macrophage colony-stimulating factor; granulocyte colony-stimulating factor, and interleukin-3) on phagocyte oxidative activity. *Blood* **81**: 1863–1870

Szamel M, Leufgen H, Kurrle R and Resch K (1995) Differential signal transduction pathways regulating interleukin-2 sysnthesis and interleukin-2 receptor expression in stimulated human lymphocytes. *Biochim Biophys Acta* **1235**: 33–42

Terashima T, Soejima K, Waki Y, Nakamura H, Fujishima S, Suzuki Y, Ishizaka A and Kanazawa M (1995) Neutrophils activated by granulocyte colony-stimulating factor suppress tumor necrosis factor-alpha release from monocytes stimulated by endotoxin. *Am J Respir Cell Mol Biol* **13**: 69–73

Tsuji T, Nagata K, Koike J, Todoroki N and Irimura T (1994) Induction of superoxide anion production from monocytes an neutrophils by activated platelets through the P-selectin-sialyl Lewis X interaction. *J Leukoc Biol* **56**: 583–587

Vechiarelli A, Monari C, Baldelli F, Pietrella D and Retini C (1995) Beneficial effect of recombinant human granulocyte colony-stimulating factor on fungicidal activity of polymorphonuclear leukocytes from patients with AIDS. *J Infect Dis* **171**: 1448–1454

Welte K, Gabrilove J, Bronchud M, Platzer E and Morstyn G (1996) Filgrastim (r.-metHuG-CSF): the first 10 years. *Blood* **88**: 1907–1929

© Cancer Research Campaign 1999
